# Supplementary material for: Maternal educational level and preterm birth: Exploring inequalities in a hospital-based cohort study
Source: PLoS One. 2023 Apr 5;18(4):e0283901. doi: 10.1371/journal.pone.0283901 (PMC10075484; doi:10.1371/journal.pone.0283901)
Supplement: S1 Table — RR: Relative Risk; adj: Adjusted for maternal age, maternal region of birth and neighbourhood deprivation index; 95%CI: 95% confidence interval. (PDF) [file pone.0283901.s001.pdf]

S1 Table. RR of spontaneous preterm birth, iatrogenic preterm birth, preterm birth before 34 weeks of gestational age and preterm birth from 34 weeks of gestational age according to educational level, maternal health conditions and health-related behaviours

	Spontaneous preterm birth		Iatrogenic preterm birth		Less than 34 weeks		34 weeks or more	
	RR adj	(95% CI)	RR adj	(95% CI)	RR adj	(95% CI)	RR adj	(95% CI)
Educational level								
High	1.00	(Ref)	1.00	(Ref)	1.00	(Ref)	1.00	(Ref)
Medium	1.35	(0.98-1.87)	1.18	(0.80-1.74)	1.37	(0.88-2.13)	1.24	(0.92-1.68)
Low	1.83	(1.25-2.68)	1.28	(0.79-2.07)	1.77	(1.07-2.94)	1.53	(1.06-2.21)
Body mass index								
Underweight	1.18	(0.65-2.12)	1.17	(0.51-2.66)	0.19	(0.03-1.37)	1.69	(1.02-2.78)
Normal weight	1.00	(Ref)	1.00	(Ref)	1.00	(Ref)	1.00	(Ref)
Overweight	1.22	(0.88-1.70)	1.94	(1.32-2.86)	1.12	(0.71-1.75)	1.67	(1.24-2.25)
Obese	1.24	(0.78-1.96)	2.42	(1.50-3.90)	0.89	(0.44-1.78)	2.10	(1.45-3.05)
Diabetes	1.21	(0.83-1.78)	1.23	(0.77-1.95)	0.59	(0.30-1.15)	1.60	(1.15-2.22)
Hypertension	0.74	(0.28-1.97)	2.28	(1.13-4.60)	3.07	(1.65-5.77)	0.36	(0.89-1.42)
Anemia	1.21	(0.71-2.04)	2.65	(1.66-4.21)	2.44	(1.47-4.06)	1.38	(0.86-2.24)
Preeclampsia	2.28	(1.45-3.58)	12.9	(9.53-17.4)	6.66	(4.49-9.87)	5.38	(4.02-7.21)
Genitourinary tract infection	17.13	(13.4-21.9)	6.43	(3.53-11.7)	33.0	(24.6-44.2)	5.22	(3.25-8.38)
Smoking	1.59	(1.16-2.19)	1.54	(1.01-2.33)	1.25	(0.78-2.01)	1.72	(1.28-2.32)
Alcohol consumption	1.00	(0.25-3.95)	2.43	(0.78-7.52)	0.89	(0.13-6.35)	1.85	(0.71-4.83)
Drugs consumption	5.11	(2.72-9.60)	6.28	(2.85-13.8)	4.86	(1.85-12.8)	5.62	(3.21-9.85)
Inadequate prenatal care	2.60	(1.55-4.36)	0.87	(0.28-2.66)	0.88	(0.27-2.84)	2.58	(1.55-4.28)

RR: Relative Risk; adj: Adjusted for maternal age, maternal region of birth and neighbourhood deprivation index

95% CI: 95% confidence interval
