# Supplementary material for: Maternal educational level and preterm birth: Exploring inequalities in a hospital-based cohort study
Source: PLoS One. 2023 Apr 5;18(4):e0283901. doi: 10.1371/journal.pone.0283901 (PMC10075484; doi:10.1371/journal.pone.0283901)
Supplement: S2 Table — RR: Relative Risk; adj: Adjusted for maternal age, maternal region of birth and neighbourhood deprivation index; BMI: body mass index; % change = [(RR without the mediator–RR with the mediator) / (1- RR without the mediator) x 100]. (PDF) [file pone.0283901.s002.pdf]

S2 Table. RR for the associations between maternal educational level and different groups of preterm birth (spontaneous, iatrogenic, less than 34 weeks and 34 weeks or more) before and after including intermediate variables and calculation of the percentage of change in the RR

Spontaneous preterm birth

	RR adj without intermediate variables	RR adj, BMI included	RR adj, smoking included	RR adj, alcohol consumption included	RR adj, drugs consumption included	RR adj, infection included
**Educational level**						
High	1.00 (Ref)	1.00 (Ref)	1.00 (Ref)	1.00 (Ref)	1.00 (Ref)	1.00 (Ref)
Secondary	1.35 (0.98-1.87)	1.30 (0.92-1.85)	1.29 (0.94-1.77)	1.35 (0.98-1.86)	1.30 (0.94-1.80)	1.33 (0.97-1.83)
% change		-14.3	-17.1	0.0	-14.3	-5.7
Primary or less	1.83 (1.25-2.68)	1.75 (1.13-2.70)	1.72 (1.09-2.18)	1.82 (1.24-2.68)	1.73 (1.18-2.53)	1.70 (1.15-2.51)
% change		-9.6	-13.3	-1.2	-12.0	-15.7

	RR adj, prenatal care included	RR adj, preeclampsia included	RR adj, diabetes included	RR adj, anemia included	RR adj, hypertension included	RR adj, all variables included
**Educational level**						
High	1.00 (Ref)	1.00 (Ref)	1.00 (Ref)	1.00 (Ref)	1.00 (Ref)	1.00 (Ref)
Secondary	1.34 (0.97-1.85)	1.32 (0.96-1.83)	1.34 (0.97-1.86)	1.35 (0.97-1.86)	1.34 (0.98-1.87)	1.18 (0.83-1.66)
% change	-2.9	-8.6	-2.9	0.0	-2.9	-48.6
Primary or less	1.79 (1.23-2.62)	1.79 (1.22-2.62)	1.81 (1.24-2.66)	1.82 (1.24-2.66)	1.83 (1.25-2.68)	1.39 (0.91-2.15)
% change	-4.8	-4.8	-2.4	-1.2	0.0	-53.0

Iatrogenic preterm birth

	RR adj without intermediate variables	RR adj, BMI included	RR adj, smoking included	RR adj, alcohol consumption included	RR adj, drugs consumption included	RR adj, infection included
**Educational level**						
High	1.00 (Ref)	1.00 (Ref)	1.00 (Ref)	1.00 (Ref)	1.00 (Ref)	1.00 (Ref)
Secondary	1.18 (0.80-1.74)	0.99 (0.65-1.50)	1.14 (0.77-1.69)	1.18 (1.00-1.63)	1.15 (0.77-1.70)	1.17 (0.79-1.73)
% change		-105.6	-22.2	0.0	-16.7	-5.6

Primary or less	1.28 (0.79-2.07)	0.94 (0.55-1.61)	1.23 (0.76-1.99)	1.27 (0.79-2.05)	1.23 (0.77-1.97)	1.24 (0.76-2.00)
% change		-121.4	-17.9	-3.6	-17.9	-14.3

	**RR adj, prenatal care included**	**RR adj, preeclampsia included**	**RR adj, diabetes included**	**RR adj, anemia included**	**RR adj, hypertension included**	**RR adj, all variables included**
**Educational level**						
High	1.00 (Ref)	1.00 (Ref)	1.00 (Ref)	1.00 (Ref)	1.00 (Ref)	1.00 (Ref)
Secondary	1.19 (0.80-1.75)	1.06 (0.73-1.55)	1.17 (0.79-1763)	1.18 (0.80-1.73)	1.17 (0.79-1.72)	0.91 (0.61-1.36)
% change	5.6	-66.7	-5.6	0.0	-5.6	-150.0
Primary or less	1.30 (0.81-2.11)	1.20 (10.75-1.91)	1.26 (0.77-2.04)	1.25 (0.78-2.03)	1.28 (0.79-2.06)	0.90 (0.54-1.51)
% change	7.1	-28.6	-7.1	-10.7	0.0	-135.7

Less than 34 weeks of gestational age

	**RR adj without intermediate variables**	**RR adj, BMI included**	**RR adj, smoking included**	**RR adj, alcohol consumption included**	**RR adj, drugs consumption included**	**RR adj, infection included**
**Educational level**						
High	1.00 (Ref)	1.00 (Ref)	1.00 (Ref)	1.00 (Ref)	1.00 (Ref)	1.00 (Ref)
Secondary	1.37 (0.88-2.13)	1.10 (0.67-1.82)	1.35 (0.87-2.10)	1.38 (0.88-2.14)	1.33 (0.86-2.06)	1.34 (0.89-2.04)
% change		-73.0	-5.4	2.7	-10.8	-8.1
Primary or less	1.77 (1.07-2.94)	1.40 (0.77-2.56)	1.74 (1.04-2.88)	1.79 (1.08-2.97)	1.70 (1.03-2.81)	1.53 (0.90-2.60)
% change		-48.1	-3.9	2.6	-9.1	-31.2

	**RR adj, prenatal care included**	**RR adj, preeclampsia included**	**RR adj, diabetes included**	**RR adj, anemia included**	**RR adj, hypertension included**	**RR adj, all variables included**
**Educational level**						
High	1.00 (Ref)	1.00 (Ref)	1.00 (Ref)	1.00 (Ref)	1.00 (Ref)	1.00 (Ref)
Secondary	1.37 (0.88-2.14)	1.28 (0.82-1.99)	1.38 (0.89-2.14)	1.37 (0.88-2.12)	1.35 (0.87-2.09)	0.98 (0.58-1.63)
% change	0.0	-24.3	2.7	0.0	-8.1	-105.4
Primary or less	1.78 (1.08-2.95)	1.68 (1.01-2.78)	1.79 (1.08-2.97)	1.73 (1.04-2.87)	1.77 (1.07-2.92)	1.15 (0.61-2.15)
% change	1.3	-11.7	2.6	-5.2	0.0	-80.5

34 weeks of gestational age or more

	RR adj without intermediate variables	RR adj, BMI included	RR adj, smoking included	RR adj, alcohol consumption included	RR adj, drugs consumption included	RR adj, infection included
**Educational level**						
High	1.00 (Ref)	1.00 (Ref)	1.00 (Ref)	1.00 (Ref)	1.00 (Ref)	1.00 (Ref)
Secondary	1.24 (0.92-1.68)	1.19 (0.87-1.63)	1.18 (0.87-1.59)	1.24 (0.91-1.68)	1.21 (0.89-1.63)	1.24 (0.91-1.67)
% change		-20.8	-25.0	0.0	-12.5	0.0
Primary or less	1.53 (1.06-2.21)	1.37 (0.92-2.05)	1.43 (0.99-2.05)	1.51 (1.04-2.19)	1.45 (1.01-2.09)	1.47 (1.02-2.13)
% change		-30.2	-18.9	-3.8	-15.1	-11.3
	**RR adj, prenatal care included**	**RR adj, preeclampsia included**	**RR adj, diabetes included**	**RR adj, anemia included**	**RR adj, hypertension included**	**RR adj, all variables included**
**Educational level**						
High	1.00 (Ref)	1.00 (Ref)	1.00 (Ref)	1.00 (Ref)	1.00 (Ref)	1.00 (Ref)
Secondary	1.24 (0.91-1.67)	1.17 (0.87-1.59)	1.23 (0.91-1.66)	1.24 (0.92-1.68)	1.25 (0.92-1.69)	1.06 (0.78-1.45)
% change	0.0	-29.2	-4.2	0.0	0.0	-75.0
Primary or less	1.51 (1.05-2.18)	1.45 (1.00 (Ref)-2.09)	1.50 (1.03-2.17)	1.52 (1.05-2.20)	1.53 (1.06-2.22)	1.15 (0.78-1.70)
% change	-3.8	-15.1	-5.7	-1.9	0.0	-71.7

RR: Relative Risk; adj: Adjusted for maternal age, maternal region of birth and neighbourhood deprivation index: BMI: body mass index

% change = [(RR without the mediator – RR with the mediator) / (1- RR without the mediator) x 100]
